# CNS efficacy parameters of combination antiretroviral therapy in chronic HIV infection: A multi-voxel magnetic resonance spectroscopy study

**DOI:** 10.3389/fneur.2023.943183

**Published:** 2023-03-24

**Authors:** Snezana Brkic, Benjamin Veres, Majda M. Thurnher, Jasmina Boban, Bojan Radovanovic, Slavica Tomic, Dusko Kozic

**Affiliations:** ^1^Faculty of Medicine, University of Novi Sad, Novi Sad, Serbia; ^2^Department of Biomedical Imaging and Image-guided Therapy, Medical University of Vienna, Vienna, Austria

**Keywords:** highly active antiretroviral therapy, magnetic resonance spectroscopy, HIV infection, treatment efficacy, AIDS

## Abstract

This study aimed to determine the correlations of combination antiretroviral therapy (cART) efficacy parameters in the central nervous system (CNS) with a neurometabolic profile on magnetic resonance spectroscopy (MRS) in virally suppressed, neurologically asymptomatic HIV+ individuals. In total, 32 HIV+ individuals on stable cART with an average age of 41.97 ± 10.12 years and with available clinical data, CNS penetration effectiveness (CPE), and monocyte efficacy (ME) scores underwent multi-voxel MRS. The parameters of neuronal number/function (NAA/Cr), membrane turnover (Cho/Cr), and glial proliferation (mI/Cr) were analyzed in supratentorial white and gray matter. Correlations of CPE and ME with neurometabolic ratios were performed using Pearson's correlation test. Statistical significance was set at *p* < 0.05. A strong positive correlation was observed between Cho/Cr and CPE in the left parietal subcortical white matter (*r* = 0.577, *p* = 0.001). A strong positive correlation between NAA/Cr and ME was obtained in the left (*r* = 0.521, *p* = 0.003) and the right (*r* = 0.494, *p* = 0.005) posterior cingulate. A strong negative correlation between ME and Cho/Cr ratios was observed in the right frontal deep white matter (*r* = −0.569, *p* = 0.001). Indices designed to assess cART efficacy in CNS failed to present significant correlations with the neurometabolic profile obtained using MRS. There is a need to define more potent non-invasive tools for neuroinflammation assessment given the prolonged life expectancy in the HIV+ population.

## Introduction

The introduction of combination antiretroviral therapy (cART) in the treatment of human immunodeficiency virus (HIV) infection has significantly decreased both the mortality and the incidence of HIV-related complications (1). It successfully suppresses the replication of HIV and thus prevents immunological and clinical deterioration of the infected individual (2). However, the incidence of mild forms of HIV-associated neurocognitive disorder (HAND)—asymptomatic neurocognitive disorder and mild cognitive disorder—has not significantly decreased, despite peripherally successful cART (3). HAND is related to lower adherence and decreased everyday functioning abilities.

The diagnosis of HAND is based on clinical examination, neurocognitive testing (according to the Frascati criteria), and neuroimaging methods (3). Clinical examination and laboratory data (including plasma viral load and immune system parameters) have low sensitivity and specificity for HIV-related neuronal injury. Peripherally successful cART and a viral load under the detection limit do not guarantee an identical situation in the central nervous system (CNS) compartment, due to a phenomenon referred to as “viral escape” (4). HIV can cross the hematoencephalic barrier (HEB) directly—*via* adsorptive transcytosis—and indirectly—nested in infected monocytes (5). HIV-infected monocytes cross the HEB and induce the process of progressive low-level neuroinflammation, which finally results in neurodegeneration caused by neuronal loss and/or dysfunction. These processes are suggested to be the basis of HAND development (6). Since the replication of HIV in neurons is not possible, HIV-related neuronal injury is probably caused by direct neurotoxicity of the virus *per se* and by indirect complex mechanisms (oxidative stress, mitochondrial dysfunction, and influx of Ca^++^ in the cell) (7–9).

Recent neuroimaging studies showed several important characteristics of brain injury in chronic HIV infection: global parenchymal reduction (loss of both gray and white matter), changes in the neurometabolic profile, and disturbances in neurotransmission (10, 11). Studies based on magnetic resonance spectroscopy (MRS) showed ongoing inflammation and progressive neurodegeneration, even in virally suppressed, neurologically asymptomatic HIV-infected individuals with normal findings on conventional magnetic resonance imaging (MRI) (10, 12). MRS is a non-invasive diagnostic modality that enables *in vivo* monitoring of subtle changes in the neurometabolic profile and has a potential role in the evaluation of cART efficacy in the CNS compartment.

Currently, there are two clinically used parameters for the assessment of cART efficacy in the brain. One is the CNS penetration effectiveness (CPE) score, introduced in 2008 and revised in 2010 (13). Values ranging from 1 to 4 are attributed to each antiretroviral drug in a combination regimen, and the score is obtained by the simple addition of those values. Due to the uncertain predictive value of the CPE score, a new scoring system was introduced recently, called the monocyte efficacy (ME) score (14), and based on the fact that monocytes are the main transporting cells of HIV to the brain. However, both of these currently used parameters seem not to reflect the exact state of neuroinflammation processes in the brain in well-controlled, chronic HIV infection. Thus, the classification of a cART regimen as “a CNS effective one” is rather controversial. We hypothesized that neither of these two indices will correlate with the neurometabolic profile reflecting low-level neuroinflammation in the brain of HIV-positive persons on otherwise successful cART, obtained on MR spectroscopy.

This study aimed to evaluate the correlations between CPE and ME scores, and the neurometabolic profile obtained by multi-voxel MRS in virally suppressed, neurologically asymptomatic, chronically infected HIV-positive individuals under cART.

## Materials and methods

### Subject selection

Initially, 41 patients were randomized for the study. Later, after exclusions, a total of 32 HIV-positive patients remained, of which 28 (87.5%) were men and 4 (12.5%) were women, with an average age of 41.97 ± 10.12 years (range 25–61). They were enrolled for this institutional ethical board-approved study carried out between October 2019 and December 2020. The dominant way of transmission was *via* sexual intercourse (mainly MSM (men having sex with men) population). Clinical data for participants (nadir CD4+ count, current CD4+ count, plasma viral load dynamics, and cART regimen) were obtained from medical history or electronic medical records. The nadir CD4+ count represents the lowest CD4+ cell count in the patient's history, usually observed at the time of HIV diagnosis. Data about the duration of HIV infection and education level were obtained per anamnesis. CPE and ME scores were calculated for each participant, given that each participant was under the same regimen of cART for at least 1 year. The revised CPE score calculation method was used in the following manner: each drug is attributed a certain value between 0 (zero) and 4 (four, which is the highest possible value) describing the CNS penetration, and the score is obtained by summing up all the values for the drugs used in the individual cART regimen (15). ME score was calculated according to the algorithm proposed by Shikuma et al.: the ME score for individual cART regimens is expressed as summed reciprocal score of each drug median effective concentration (EC_50_) based on the acute infection model (14). All subjects were virally suppressed, with plasma viral loads under the limit of detection (< 40 copies/mL) for at least 1 year, based on polymerase chain reaction (PCR) testing (RealTime Abbot). The immune status of all participants was reconstituted, with the current CD4+ cell count in all patients being higher than 200 cells/mL. All subjects underwent fast screening neurocognitive testing, International HIV Dementia Scale (IHDS), for the elimination of HIV-associated dementia.

The inclusion criteria were as follows: age over 18 years, HIV-positive status (based on PCR testing), adequate peripheral viral suppression, current CD4+ count over 200, unchanged cART regimen for at least 1 year, and normal conventional MR imaging (no morphologic abnormalities observed on standard native MRI of the brain). Exclusion criteria were as follows: the presence of opportunistic infections of CNS, coinfection with hepatitis C or B, intravenous drug abuse, major psychiatric disorders including major depression, chronic neurological disease (ischemic, vasculitis, infiltrative, and multiple sclerosis), the presence of systemic disease (autoimmune disorders), and the International HIV Dementia Score (IHDS) score ≤ 10 (the maximum score is 12, every subject that scores < 10 should undergo further evaluation for potential dementia).

The study was approved by the institutional ethical committee and was performed in accordance with all relevant guidelines and regulations. All subjects signed informed consent for participation in the study (according to the Declaration of Helsinki).

### Imaging protocol

All participants underwent a standard brain imaging protocol on a 3T MR scanner (Trio Tim, Siemens, Erlangen, Germany), using an 8-channel head array coil. It consisted of sagittal T1-weighted images, axial T2-weighted images, FLuid Attenuation Recovery (FLAIR), coronal T2-weighted images, and diffusion-weighted imaging (DWI). Standard brain imaging was performed to exclude focal and diffuse lesions of white and gray matter and to enable voxel positioning. The exclusion criteria for MRI findings in the brain were mentioned earlier, consisting of any focal or diffuse abnormalities of the signal intensity, including various focal lesions associated with infection, inflammation, tumors, vascular malformations, congenital abnormalities, white matter hyperintensities (WMHs), and also diffuse abnormalities such as global reduction of the brain volume not proportional to the age of the participant.

**Figure 1:**
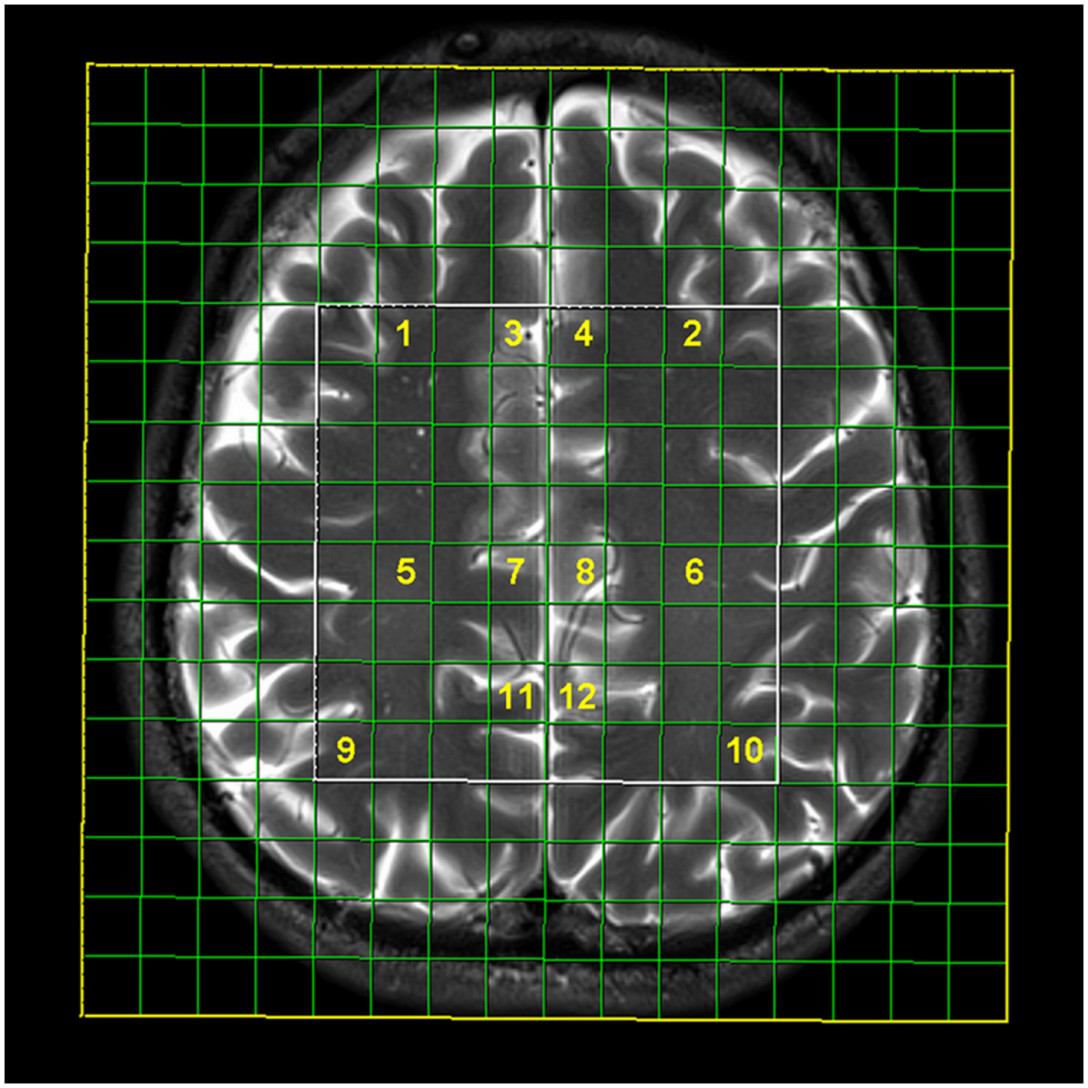
Multi-voxel magnetic resonance spectroscopy network with selected localizations: frontal subcortical white matter on the right (1) and left (2), anterior cingulate gyrus on the right (3, 7) and left (4, 8), posterior cingulate on the right (11) and left (12), frontal deep white matter on the right (5) and left (6), and parietal subcortical white matter on the right (9) and left (10).

Two-dimensional (2D) multi-voxel Point RESolved Spectroscopy (PRESS) MRS was performed in short- (time of repetition, TR = 1,530 ms and time of echo, TE = 30 ms) and long-echo time (TR/TE 1,530 ms/135 ms) technique (scan duration 8:17 min for long-echo and 7:50 min for short-echo time MRS; FOV 160 × 160 × 160 mm, VOI 80 × 80 × 10 mm), with the placement of 8 × 8 multi-voxel network in supratentorial white and gray matter, just above the corpus callosum. The nominal resolution of the voxels was 10 × 10 × 10 mm (1 cm^3^). The number of phase encoding steps was 16 in all directions, and the interpolation resolution was 16 in all directions. Region of interest was placed identically in each participant by an experienced neuroradiologist (with over 15 years of experience), by placing the voxel grid in the supracallosal white and gray matter using the anterior cingulate-posterior cingulate gyrus line as an anatomical marker. Positions of analyzed voxels were manually chosen on the workstation (Figure 1). Spectroscopy data were analyzed in post-processing on the commercial workstation (Leonardo, Siemens Medical Systems, Erlangen, Germany); correction and calculation of mean values, identification of peaks, and ratio calculation were performed for each voxel. Typical signals that were analyzed on a spectroscopic scale were:

1. N-acetyl aspartate (NAA) at 2.02 parts per million (ppm), a marker of neuronal number and/or function.
2. Choline (Cho), at 3.2 ppm, represents the cell membrane marker.
3. Myoinositol (mI) at 3.5 ppm, glial marker and osmoregulator; present only on short-echo time MRS.
4. Creatine (Cr) at 3.0 ppm, a marker of energy reserve and a reference marker.

Ratios of NAA/Cr, Cho/Cr, and mI/Cr were calculated.

### Statistical analysis

Statistical analysis was performed using the SPSS version 23.0 software tool (IBM, Chicago, IL).

Descriptive analysis included mean values and standard deviations, or median values and interquartile ranges (as appropriate), for quantitative values. For qualitative values, frequencies and ranges were used. The correlation between CPE and ME scores and neurometabolite ratios obtained on multi-voxel MRS was examined using Pearson's correlation test; in addition, we tested correlations between CPE and ME scores. The correlation coefficient (r) ranged from −1 to 1 (depending on whether the correlation was positive or negative). The strength of the correlation was determined by absolute values, divided into poor (0.10–0.29), moderate (0.30–0.49), and strong (over 0.50) (16). The statistical value was set at *p* < 0.05. Given that multiple comparisons were performed during the statistical analysis, we added statistical rigor in the form of Holm modification of the Bonferroni method.

## Results

After excluding nine patients due to various exclusion criteria, a total of 32 patients were included in the analysis. Five patients were excluded due to detected focal abnormalities on brain imaging (mainly chronic microangiopathic lesions and white matter hyperintensities, and one patient has lacunar infarctions). Two patients were excluded due to the poor quality of spectra obtained, one patient was excluded due to the IHDS score (10), and one patient interrupted the examination before the completion of MRS. The demographic and clinical data of the study sample are summarized in Table 1. Patients were receiving the following types of cART: 3TC (lamivudine)+ABC (abacavir)+LOP/r (lopinavir/ritonavir) (six patients. 18.75%; CPE score 8, ME score 61.3), AZT (zidovudine)+ABC+LOP/r (12 patients, 37.5%; CPE 10, ME 61.3), 3TC+AZT+LOP/r (12 patients, 37.5%; CPE 9, ME 108.3), and 3TC+AZT (two patients, 6.25%; CPE 6, ME 100). The mean values of the metabolite ratios are shown in Table 2 for each analyzed voxel, separately.

**Table 1:** Clinical and demographic characteristics of the study sample.

**Variable**	***N***	**Mean**	**SD**	**Minimum**	**Maximum**
Age (years)	32	41.97	10.12	25	61
Education (years)	32	10.75	2.91	4	16
Duration of cART (years)	32	5.59	4.457	1	16
Current CD4+ count (cells/μl)	32	620.28	254.36	177	1,122
Current CD8+ count (cells/μl)	32	1072.47	475.71	79	2,000
CD4/CD8 ratio	32	1.38	3.62	0.18	21
Nadir CD4+ count (cells/μl)	32	199.28	140.13	1	487

N, number; SD, standard deviation; cART, combination antiretroviral therapy.

**Table 2:** Mean values of neurometabolite ratios in analyzed locations of the brain.

**NAA/Cr**→**Cho/Cr**→**mI/Cr**					
**Short-echo time**→**Long-echo time**→**Short-echo time**→**Long-echo time**→**Short-echo time**					
Right FSWM	1.78 ± 0.6	1.68 ± 0.36	1.08 ± 0.25	1.04 ± 0.25	0.49 ± 0.16
Right vACG	1.92 ± 0.55	1.35 ± 0.22	1.10 ± 0.15	1.02 ± 0.15	0.68 ± 0.21
Left vACG	2.36 ± 0.43	2.16 ± 0.35	1.02 ± 0.15	0.95 ± 0.22	0.47 ± 0.16
Left FSWM	1.98 ± 0.49	1.59 ± 0.19	1.03 ± 0.15	0.89 ± 0.14	0.59 ± 0.15
Right FDWM	2.40 ± 0.58	2.43 ± 0.46	0.87 ± 0.17	0.80 ± 0.20	0.39 ± 0.14
Right dACG	2.01 ± 0.43	1.98 ± 0.31	0.79 ± 0.14	0.73 ± 0.17	0.38 ± 0.12
Left dACG	1.89 ± 0.58	1.56 ± 0.30	1.04 ± 0.14	1.00 ± 0.21	0.77 ± 0.22
Left FDWM	1.91 ± 0.47	1.34 ± 0.21	1.13 ± 0.18	1.04 ± 0.18	0.69 ± 0.20
Right PSWM	2.18 ± 0.45	1.97 ± 0.34	1.06 ± 0.13	0.95 ± 0.16	0.71 ± 0.19
Right PCG	1.95 ± 0.32	1.54 ± 0.16	1.02 ± 0.12	0.91 ± 0.15	0.60 ± 0.12
Left PCG	2.18 ± 0.57	2.09 ± 0.39	0.86 ± 0.25	0.77 ± 0.19	0.47 ± 0.18
Left PSWM	2.17 ± 0.44	1.97 ± 0.27	0.81 ± 0.15	0.72 ± 0.17	0.41 ± 0.11

FSWM, frontal subcortical white matter; vACG, ventral anterior cingulate gyrus; dACG, dorsal anterior cingulate gyrus; FDWM, frontal deep white matter; PCG, posterior cingulate gyrus; PSWM, parietal subcortical white matter.

Table 3 summarizes the results of the correlation analysis between CPE scores and neaurometabolite ratios in the observed brain regions. Correlations of metabolite ratios with CPE were mainly poor. Only in several locations, intermediate and strong correlations were observed. The only region with a strong positive correlation between Cho/Cr and CPE score on long-echo time MRS was the left parietal subcortical white matter (*r* = 0.577, *p* = 0.001). When adding statistical rigor to the measurements, this result persisted as the only significant one.

**Table 3:** Correlations of CPE score and neurometabolite ratios in the observed brain regions.

**Correlation between**		**RFSWM**	**RvACG**	**LvACG**	**LFSWM**	**RFDWM**	**RdACG**	**LdACG**	**LFDWM**	**RPSWM**	**RPCG**	**LPCG**	**LPSWM**
**Short-echo MRS**													
CPE and NAA/Cr	*r*	−0.124	0.267	−0.221	−0.290	0.006	−0.088	0.330	−0.099	−0.304	0.084	−0.031	−0.133
	*p*	0.508	0.147	0.231	0.113	0.976	0.640	0.069	0.597	0.096	0.654	0.869	0.475
CPE and Cho/Cr	*r*	0.350	−0.046	0.091	0.224	−0.257	0.279	−0.125	0.098	0.295	0.041	0.120	0.446
	*p*	0.053	0.807	0.625	0.226	0.162	0.129	0.501	0.601	0.107	0.826	0.519	**0.012**
CPE and mI/Cr	*r*	−0.019	0.150	−0.197	0.096	−0.079	−0.024	0.187	0.097	0.230	−0.187	0.189	0.046
	*p*	0.921	0.420	0.289	0.609	0.672	0.897	0.313	0.604	0.214	0.314	0.309	0.805
**Long-echo MRS**													
CPE and NAA/Cr	*r*	−0.208	0.064	−0.156	−0.009	0.084	−0.086	−0.056	−0.194	−0.138	0.023	−0.030	0.181
	*p*	0.260	0.732	0.402	0.962	0.653	0.644	0.763	0.296	0.459	0.902	0.871	0.329
CPE and Cho/Cr	*r*	0.037	0.494	−0.084	0.209	0.230	0.259	0.386	−0.176	0.202	0.129	0.255	0.557
	*p*	0.845	**0.005**	0.652	0.260	0.213	0.159	**0.032**	0.342	0.277	0.487	0.167	**0.001**

RFSWM, right frontal subcortical white matter; LFSWM, left frontal subcortical white matter; RvACG, right ventral anterior cingulate gyrus; LvACG, left ventral anterior cingulate gyrus; RdACG, right dorsal anterior cingulate gyrus; LdACG, left dorsal anterior cingulate gyrus; RFDWM, right frontal deep white matter; LFDWM, left-right frontal deep white matter; RPCG, right posterior cingulate gyrus; LPCG, left posterior cingulate gyrus; LPSWM, left parietal subcortical white matter; CPE, CNS penetration effectiveness. Results in bold represent significant values, < 0.05.

Correlations between ME scores and neurometabolite ratios are presented in Table 4. Here, we obtained strong positive correlations between NAA/Cr and ME in the left (*r* = 0.521, *p* = 0.003) and the right (*r* = 0.494, *p* = 0.005) posterior cingulate gyrus. A strong negative correlation between ME and Cho/Cr ratios was observed in the right frontal deep white matter (*r* = −0.569, *p* = 0.001), and moderate negative correlations were observed in the left frontal and right parietal subcortical white matter. After adding statistical rigor to the analysis, only *p*-values < 0.005 persisted as significant. On the long-echo time MRS, negative correlations were additionally detected in the right PCG. There were no significant correlations between the ME score and the nadir CD4+ count or the current CD4+/CD8+ ratio in our study sample.

**Table 4:** Correlations between ME score and neurometabolite ratios, in analyzed brain regions (*r* is presented in the upper row and *p* in the lower row in the cell).

**Correlation between**		**RFSWM**	**RvACG**	**LvACG**	**LFSWM**	**RFDWM**	**RdACG**	**LdACG**	**LFDWM**	**RPSWM**	**RPCG**	**LPCG**	**LPSWM**
**Short-echo MRS**													
ME and NAA/Cr	R	−0.236	0.046	−0.172	0.251	−0.332	−0.424	0.010	0.079	0.372	0.013	0.521	0.485
	P	0.202	0.807	0.356	0.173	0.068	**0.018**	0.955	0.675	**0.039**	0.946	**0.003**	**0.006**
ME and Cho/Cr	R	0.044	−0.222	−0.087	−0.421	−0.374	−0.047	−0.264	−0.276	−0.378	−0.254	−0.220	−0.292
	P	0.813	0.231	0.641	**0.018**	**0.038**	0.801	0.151	0.133	**0.036**	0.167	0.233	0.111
ME and mI/Cr	R	−0.401	0.006	−0.097	0.140	−0.152	−0.161	−0.096	0.071	0.107	−0.022	−0.154	0.201
	P	**0.025**	0.974	0.605	0.453	0.414	0.386	0.606	0.705	0.568	0.908	0.409	0.279
**Long-echo MRS**													
ME and NAA/Cr	R	−0.274	−0.354	−0.070	−0.130	−0.043	−0.326	−0.255	−0.329	−0.046	−0.494	0.149	0.298
	P	0.135	**0.051**	0.707	0.484	0.820	0.074	0.166	0.071	0.804	**0.005**	0.425	0.103
ME and Cho/Cr	R	−0.149	−0.183	−0.068	−0.336	−0.569	−0.056	−0.060	−0.193	−0.306	−0.447	−0.273	−0.074
	P	0.423	0.325	0.715	0.064	**0.001**	0.765	0.747	0.299	0.095	**0.012**	0.137	0.693

RFSWM, right frontal subcortical white matter; LFSWM, left frontal subcortical white matter; RvACG, right ventral anterior cingulate gyrus; LvACG, left ventral anterior cingulate gyrus; RdACG, right dorsal anterior cingulate gyrus; LdACG, left dorsal anterior cingulate gyrus; RFDWM, right frontal deep white matter; LFDWM, left-right frontal deep white matter; RPCG, right posterior cingulate gyrus; LPCG, left posterior cingulate gyrus; LPSWM, left parietal subcortical white matter; CPE, CNS penetration effectiveness. Results in bold represent significant values, < 0.05.

The correlation between CPE and ME in our study cohort was positive but did not reach statistical significance (*r* = 0.219, *p* = 0.089).

Since previous studies implied the relationship between NAA level and brain aging, with NAA/Cr ratios progressively declining and mI, Cho, and Cr levels progressively increasing with advancing age (17, 18), correlation analysis of NAA/Cr, mI/Cr, and Cho/Cr ratios and age was performed. No significant correlations of NAA/Cr, Cho/Cr, and mI/Cr ratios with the age of the participants were observed in our study population.

## Discussion

Combination antiretroviral therapy (cART) has changed the prevalence of various types of HIV-associated neurocognitive disorders, with milder forms [asymptomatic neurocognitive impairment (ANI) and mild neurocognitive impairment (MNI)] becoming more common and present in approximately one-third of HIV-infected individuals in the cART era (1, 2). Given that the life expectancy of HIV-positive individuals has reached that of a healthy person, it is expected that milder forms of HAND will become even more prevalent in the future. One of the possible explanations might lie in the suboptimal efficacy of cART in the CNS compartment, despite peripherally successful viral suppression. Recent data confirmed the phenomenon of “viral escape,” as the consequence of cART concentrations being higher in plasma than in cerebrospinal fluid (CSF). Monitoring of drug penetration and efficacy has become an important issue in the therapy management of these patients. Letendre et al. introduced a score, named the CPE score, to indirectly assess drug penetration through the hematoencephalic barrier (HEB) (13, 15). A CPE score is calculated based on the values provided in the CNS penetration effectiveness ranks, such that every drug has a value between 0 and 4 (Letendre et al. Correlates of CSF viral loads in 1,221 volunteers of the CHARTER cohort. 17th Conference on retroviruses and opportunistic infections, USC, San Diego 2010). The score for the individual cART regimen is obtained by simply adding the values of separate drugs into a sum value. An additional (ME) score was introduced by Shikuma et al. to overcome the fact that cART suboptimally suppresses viral replication in circulating monocytes and brain macrophages, resulting in the process of unhalted neuroinflammation (14). This score is based on the anticipated efficacy of cART against monocytes using the data on *in vitro* drug efficacy. However, to date, this score was not validated for use in clinical practice. To the best of our knowledge, this is the pioneer study correlating the CNS efficacy score of cART and neurometabolic profiles obtained on MRS in chronically infected and virally suppressed HIV-positive individuals.

In this study, there were no significant correlations of the NAA/Cr and mI/Cr ratios with CPE score, both on short- and long-echo time MRS. Some positive correlations between Cho/Cr and CPE score were observed in the anterior cingulate gyrus (ACG) and the parietal subcortical white matter on the left. These findings might suggest that the higher penetration of cART in the CNS results in higher membrane turnover and gliosis. One of the explanations for this result might be that there is ongoing low-level inflammation in some regions of the brain, despite good penetration of cART. The ACG is divided into two parts, with anatomically and functionally different roles. The ventral ACG is the “emotional” part, a functional component of the limbic system, while the dorsal ACG is the “cognitive” part, important for executive functions (19). Both parts are considered to be involved in highly important “frontal lobe functions,” that are extremely sensitive to neuronal injury (20). Parietal subcortical white matter is also involved in cognitive functions (21). The basis for neurocognitive deterioration in chronic HIV infection, reflected in the disturbances observed in executive functioning, might be explained by the HIV-related injury in these particular regions. The lack of strong and significant correlations between CPE and neurometabolites is not surprising. Baker et al. failed to detect differences in cognitive functioning between the two groups of HIV-positive patients, divided by CPE score in groups with high and low drug penetrancy (22). Ellis and Letendre did not observe any improvements in cognitive functions after the switch to a highly penetrant cART, based on the CPE score (23). One must be aware that the CPE score represents the concentrations of the drugs in CSF and does not reflect the efficacy of cART inside the cells, which might explain the lack of sensitivity (14). Harezlak et al. failed to confirm any predictive value of CPE on neurometabolic scores, observed on single-voxel MRS (24).

To the best of our knowledge, no prior studies about the impact of ME on metabolite ratios in the brain were performed. Our results showed significant positive correlations between ME score and NAA/Cr levels in the right and the left posterior cingulate gyrus (PCG). The PCG is a part of the brain with high metabolic rates, a keystone of cognitive pathways, and one of the most important regions in executive functioning and memory tasks (25). Cho/Cr ratios correlated negatively with ME scores in almost all observed regions, reaching significant values mostly in the regions of white matter (frontal deep, frontal, and parietal subcortical). The only region of the gray matter where negative correlations were observed between ME scores and Cho/Cr was the right PCG, probably as a result of its high metabolic rate and vivid role in executive functioning, which is greatly disturbed during HAND. The drop in NAA/Cr was significant in the region of PCG in several recent studies (26), indicating that this specific region is the most severely affected in HIV-positive subjcets with signs of cognitive impairment. Moreover, changes in metabolic ratios in this area are suggested to be predictive of future cognitive decline in presymptomatic individuals, since the neurodegenerative process begins before manifesting cognitive decline. High ME scores in some regions of the brain imply lower membrane metabolism and lower level of gliosis, as a secondary beneficial effect of highly effective cART. In the other words, efficient cART plays an important role in halting the process of inflammation in the brain.

mI/Cr presented no significant correlations with either of the observed efficacy scores. This ratio represents the biomarker of glial proliferation and neuroinflammation and is significantly elevated in the very early course of HIV infection. However, soon after the introduction of cART, ratios of mI/Cr decline and tend to reach the level of healthy subjects (27). Since all participants in our study were under continuous cART for over 1 year, normalization of mI/Cr ratios was an expected finding. Thus, no correlations with cART penetrancy markers were observed.

The “legacy effect” represents one of the basic theories regarding the development of neurodegeneration in HIV infection, with an initial injury to neurons caused by early HIV infection. It is based on the fact that nadir CD4+ count predicts neurocognitive impairment with high accuracy (28). Nadir CD4+ count is the lowest CD4+ count in the patient's history and correlates well with the immune status. No significant correlations of nadir CD4+ count with neurometabolic parameters were observed in our study, possibly due to a small sample size (shown in Table 5).

**Table 5:** Correlations of observed brain metabolites with nadir CD4+ T-cell count in analyzed locations.

**Correlations with nadir CD4**+		**NAA/Cr (TE = 135 ms)**	**Cho/Cr (TE = 135 ms)**	**NAA/Cr (TE = 30 ms)**	**Cho/Cr (TE = 30 ms)**	**mI/Cr**
RFSWM	*r*	0.166	−0.082	0.014	−0.013	−0.154
	*p*	0.206	0.531	0.915	0.924	0.240
RvACG	*r*	0.096	0.214	−0.054	0.194	0.159
	*p*	0.584	0.118	0.681	0.138	0.246
LvACG	*r*	−0.005	0.196	0.075	0.222	0.066
	*p*	0.970	0.133	0.570	0.088	0.617
LFSWM	*r*	−0.130	−0.063	0.158	0.210	0.053
	*p*	0.346	0.632	0.227	0.107	0.689
RFDWM	*r*	0.009	0.111	0.006	−0.019	0.032
	*p*	0.948	0.400	0.966	0.883	0.808
RdACG	*r*	0.032	0.197	0.030	0.113	0.122
	*p*	0.815	0.150	0.821	0.391	0.353
LdACG	*r*	0.132	0.124	−0.056	0.089	0.259
	*p*	0.340	0.347	0.671	0.498	0.056
LFDWM	*r*	0.262	0.037	0.158	0.034	−0.198
	*p*	0.053	0.777	0.228	0.796	0.130
RPSWM	*r*	0.151	0.097	0.242	0.098	0.143
	*p*	0.271	0.461	0.062	0.456	0.413
RPCG	*r*	0.178	−0.079	0.207	0.018	−0.225
	*p*	0.192	0.549	0.113	0.894	0.083
LPCG	*r*	0.014	−0.101	0.052	0.191	−0.238
	*p*	0.915	0.441	0.695	0.144	0.067
LPSWM	*r*	0.014	0.189	−0.040	0.048	−0.237
	*p*	0.915	0.148	0.762	0.716	0.069

RFSWM, right frontal subcortical white matter; LFSWM, left frontal subcortical white matter; RvACG, right ventral anterior cingulate gyrus; LvACG, left ventral anterior cingulate gyrus; RdACG, right dorsal anterior cingulate gyrus; LdACG, left dorsal anterior cingulate gyrus; RFDWM, right frontal deep white matter; LFDWM, left-right frontal deep white matter; RPCG, right posterior cingulate gyrus; LPCG, left posterior cingulate gyrus; LPSWM, left parietal subcortical white matter.

The age of the sample is an important issue in the evaluation of the neurocognitive status of HIV- positive individuals, based on prior studies indicating the acceleration of physiological aging of the brain in controlled HIV infection (29). Aging leads to a decline in NAA/Cr levels (17). However, in our study, no significant impact of age on NAA/Cr ratios was observed. In our opinion, the reason is the study sample selection with no patients of advanced age included (only one patient was older than 55 years of age). In this age group, no significant effects of physiological aging on NAA levels are expected to be observed. Some recent studies showed an age-dependent increase in mI, Cho, and Cr levels in some regions of the brain (18, 30), primarily the dorsolateral prefrontal cortex, anterior cingulate gyrus, hippocampus/parahippocampal region, and thalamus. In our study, these age-related effects on glia-associated neurometabolites were not detected. In our opinion, the reasons not to show this effect are primarily due to locations observed (we did not include the hippocampus or basal ganglia in analysis) and the design of MRS examination, since the majority of the available data are based on single-voxel design and very long TR (< 4 s) (18). In addition, the study by Lind et al. was performed on 7T (30).

The conclusion of the CHARTER study was that numerous factors can influence the occurrence and development of HAND in chronically infected HIV-positive individuals under cART (1). Bladowska et al. showed in their recent study that HIV-positive subjects on cART have the most pronounced reduction of NAA/Cr, compared to therapy-naïve HIV-positive subjects and HIV-HCV coinfected subjects (26). It is interesting that, particularly in this study, HIV-HCV coinfected subjects showed a significant decrease of NAA/Cr in the ACG, the region where a significant correlation of metabolite ratios with CP scores was observed in our study. This might speak in favor of regional susceptibility to neurometabolic changes in chronic treated HIV infection. Co-infection with hepatitis C doubles the risk for HAND development (31), while the use of psychoactive substances (methamphetamine) led to rapid cognitive deterioration (32). Due to these results, patients with a history of drug abuse and hepatitis B and C co-infection were excluded from the study.

Finally, an important issue that has to be taken into account is the potential long-term neurotoxic effect of some antiretroviral drugs. The prolonged lifespan of HIV-positive individuals extends the period of exposure to those drugs. Neurotoxicity is caused by direct and indirect mechanisms. The indirect effect is related to vascular injury observed in patients treated with protease inhibitors, leading to atherosclerotic changes and associated vascular pathology, leading to accelerated brain aging and deposition of β-amyloid in the brain (17, 26). One additional side effect of cART that is increasingly gaining importance is the metabolic impact secondary to weight gain. Metabolic syndrome itself has a certain, yet not completely explained, contribution to the development of neuroinflammation that also lies in the center of the neuropathological process in chronic HIV infection (33). Direct toxicity was primarily observed in peripheral nerves, and later in the CNS, on animal models. Nucleoside reverse transcriptase inhibitors induce mitochondrial dysfunction (34). Some authors implied that higher CPE led to higher drug concentrations in CNS and accentuated neurotoxicity (35). Positive correlations between Cho/Cr and CPE scores observed in our sample might support this theory.

The way that cART affects the clinical outcome in patients with chronic HIV infection with regard to HAND development is controversial, given that both drug efficacy and drug neurotoxicity must be observed as synchronous processes (36). Although studies showed a lower proportion of HIV- positive individuals with detectable CSF HIV RNA among those who were taking high-CPE cART regimens (37), some authors reported that the risk for HIV-associated dementia development was increased in patients with similar highly penetrant regimens (23). However, in the clinical setting, usually the simplest, most potent, and least toxic agents are used, without paying much attention to the CPE scores. One also must be aware of the fact that our study sample was receiving a so-called “old-fashioned” cART. Recent studies showed that some modern antiretroviral drugs, such as dolutegravir, have better penetrancy into the CSF compartment and contribute to the rapid clearance of HIV from the CSF compartment (and indirectly from CNS) (38). This drug is a potent, second-generation integrase inhibitor that prevents the intergration of proviral DNA in the genome of the host (39). Further research based on animal models and clinical trials should be directed into discovering a composite biomarker of drug potency in the CNS to lower the risk of HAND development in otherwise well-controlled HIV infection.

### Strengths and limitations

As mentioned before, this is the first study to correlate between the indices of CNS efficacy of the cART regimen and neurometabolites in chronic HIV-positive subjects with successful peripheral viral suppression. It was performed on a 3T clinical scanner with strict inclusion and exclusion parameters that allowed for the homogenization of the sample included. Only patients with completely normal brain imaging were included in the study, to avoid the contamination of the spectra and biased results. In addition, we used multi-voxel MRS which allowed for a more global synchronous analysis of more regions in the brain.

Some limitations have to be mentioned. First of all, the study sample was not large, but it almost reached the minimal sample based on the power calculation (for power 0.8, alpha 0.05, and the minimum sample size was 39). Nevertheless, the correlations shown are mostly moderate and not present in the majority of observed locations, which might point to the necessity of involving more subjects in the sample or applying strict statistical corrections such as Bonferroni. An additional issue would be the inclusion of healthy controls. However, based on our previous studies (10, 12), there are significant changes in neurometabolites' ratios between HIV-positive subjects, even on successful and long-standing cART, compared to age and education-level matched controls. Since the correlations between cART efficacy parameters in healthy controls cannot be evaluated in controls, the authors felt that the inclusion of the controls would not be methodologically relevant. The last limitation would be the lack of detailed neuropsychological assessment of the subjects, including the assessment of the life quality and depression/ anxiety, which all could have a significant impact on ratios of neurometabolites.

## Conclusion

Clinically used scores designed to assess antiretroviral drug efficacy in the central nervous system compartment failed to present significant correlations with neurometabolite ratios obtained using multi-voxel MR spectroscopy. CPE score only showed a positive correlation with the marker of membrane metabolism in the parietal subcortical white matter, while there were no significant correlations with markers of neuronal function and glial proliferation. ME presented significant positive correlations with neuronal markers only in the dorsal anterior cingulate, posterior cingulate gyrus, and parietal subcortical white matter, thus indirectly confirming the role of cART in halting the process of progressive neurodegeneration in metabolically active brain regions involved in high cognitive functioning. In addition, ME showed negative correlations with the marker of membrane metabolism and gliosis, thus implying a reduced level of inflammation due to good drug penetration into monocytes. Since these biomarkers do not seem to be clinically useful either solely or in combination, more potent tools are needed to assess the level of neuroinflammation, especially given that the HIV-positive population has an extended life expectancy due to potent modern regimens of cART. A new “kick and kill” strategy in HIV treatment will open a new field for defining biomarkers of therapy efficacy and monitoring neurological side effects.

## Data availability statement

The raw data supporting the conclusions of this article will be made available by the authors, without undue reservation.

## Ethics statement

The studies involving human participants were reviewed and approved by Ethical Committee of Faculty of Medicine Novi Sad. The patients/participants provided their written informed consent to participate in this study.

## Author contributions

Conception and design and drafting the article: SB and JB. Acquisition of the data: BV and BR. Data analysis and interpretation: MT, BV, JB, BR, and ST. Revising the article: SB, DK, and MT. All authors have seen and approved the final version and have contributed significantly to the study.
